# Perturbations of Fibroblast Growth Factors 19 and 21 in Type 2 Diabetes

**DOI:** 10.1371/journal.pone.0116928

**Published:** 2015-02-09

**Authors:** Stephen L. Roesch, Amanda M. Styer, G. Craig Wood, Zachary Kosak, Jamie Seiler, Peter Benotti, Anthony T. Petrick, Jon Gabrielsen, William E. Strodel, Glenn S. Gerhard, Christopher D. Still, George Argyropoulos

**Affiliations:** 1 Institute of Obesity, Geisinger Health System, Danville, PA, United States of America; 2 Department of Surgery, Geisinger Health System, Danville, PA, United States of America; 3 Department of Biochemistry and Molecular Biology and Department of Pathology and Laboratory Medicine, Pennsylvania State University, Hershey, PA, United States of America; IRCCS Istituto Oncologico Giovanni Paolo II, ITALY

## Abstract

Fibroblast growth factors 19 and 21 (FGF19 and FGF21) have been implicated, independently, in type 2 diabetes (T2D) but it is not known if their circulating levels correlate with each other or whether the associated hepatic signaling mechanisms that play a role in glucose metabolism are dysregulated in diabetes. We used a cross-sectional, case/control, experimental design involving Class III obese patients undergoing Roux-en-Y bariatric surgery (RYGB), and measured FGF19 and FGF21 serum levels and hepatic gene expression (mRNA) in perioperative liver wedge biopsies. We found that T2D patients had lower FGF19 and higher FGF21 serum levels. The latter was corroborated transcriptionally, whereby, *FGF21,* as well as *CYP7A1, β-Klotho, FGFR4, HNF4α*, and glycogen synthase, but not of *SHP* or *FXR* mRNA levels in liver biopsies were higher in T2D patients that did not remit diabetes after RYGB surgery, compared to T2D patients that remitted diabetes after RYGB surgery or did not have diabetes. In a Phenome-wide association analysis using 205 clinical variables, higher FGF21 serum levels were associated with higher glucose levels and various cardiometabolic disease phenotypes. When serum levels of FGF19 were < 200 mg/mL and FGF21 > 500 mg/mL, 91% of patients had diabetes. These data suggest that FGF19/FGF21 circulating levels and hepatic gene expression of the associated signaling pathway are significantly dysregulated in type 2 diabetes.

## Introduction

Past studies have shown that fibroblast growth factors 19 and 21 (FGF19 and FGF21) play a role in insulin sensitivity, glucose disposal, and lipid parameters [1,2]. FGF19 and FGF21 are integrators of bile acid production and glucose metabolism in the liver [3,4]. FGF19 is stimulated in the intestine by bile acids (BA) [5] via the farnesoid x receptor (FXR) [6]. In turn, FGF19 signals through fibroblast growth factor receptor 4 (FGFR4) and βKlotho in hepatocytes to inhibit expression of the cholesterol 7 alpha-hydroxylase (CYP7A1) gene [7], which is the rate limiting enzyme for bile acid synthesis.

FGF19 has insulin-like actions and is secreted from the small intestine in response to feeding [8], while, FGF21 is secreted from the liver in response to extended fasting [9]. FGF19 and bile acids increase after RYGB surgery particularly in patients that experience diabetes remission [10]. Circulating FGF21 levels also increase after RYGB surgery [11] but it is not known if this outcome is also specific to patients that do not enter into diabetes remission. FGF19 and FGF21 may use overlapping or separate pathways of action depending on the condition and the site of action [3,12]. Yet, there is no evidence of an interaction between FGF19 and FGF21 although it has been suggested that FGF19 may directly regulate FGF21 in the liver [13].

In the present study, we tested the hypothesis that circulating levels of FGF19 and FGF21 correlate with each other, and that along with genes regulating hepatic pathways of bile acid synthesis are dysregulated in diabetes and in particular in patients that fail to remit diabetes after RYGB surgery. To test this hypothesis, we used a cohort of Class III obese patients undergoing RYGB surgery and compared FGF19 and FGF21 serum levels between diabetic and non-diabetic patients prior to surgery. We also performed a phenotype- or phenome-wide association (PheWAS) analysis by using 205 clinical variables and preoperative FGF19/21 serum levels. In addition, using liver wedge biopsies taken during surgery, we compared the expression levels of FGF21 and of key genes in the FGF19-BA pathway between diabetic and non-diabetic patients and also between diabetic patients that remit or do not remit diabetes after RYGB surgery.

## Research Design and Methods

### Study participants

The cohort used in this study consisted of Class III obese patients from Geisinger Clinic’s bariatric surgery program with a mean body mass index (BMI) of 49.6 kg/m^2^ [14]. Patients were stratified according to their diabetes status. 66 patients represented the non-diabetes (NoT2D) group and 62 patients represented the diabetes (T2D) group (**Table A in S1 File**). The T2D group was further stratified according to diabetes remission status following Roux-en-Y gastric bypass (RYGB) surgery (**Table B in S1 File**).

These studies were approved by the Geisinger Clinic Institutional Review Board for research. All participants provided written informed consent.

### Definition of type 2 diabetes and remission of type 2 diabetes

The definition of type 2 diabetes was according to ADA-recommended guidelines [15]. Diabetes was defined by fasting glucose > 126 mg/dL or HbA1c > 6.5%. The status of non-diabetes was further ascertained by the absence of diabetes medication and diagnosis of diabetes, as documented in our electronic medical records (EMR).

Remission of diabetes was defined according to the established definition of a cure of diabetes [16]. Diabetic patients were considered to be in partial or complete remission of diabetes (T2D-R) if they were free of any use of anti-diabetic medications, their fasting blood glucose levels were < 125 mg/dL for partial remission (or < 100 mg/dL for complete remission) and HbA1c was < 6.5% for partial remission (or < 5.6% for complete remission), for a minimum of 12 months after RYGB. Additional confirmation was obtained by examining their EMR for the ICD10 diagnostic code for diabetes.

### FGF19 and FGF21 serum levels

All the blood draws were obtained in the fasted state (minimum of 12-hour fast), approximately, 2 months prior to surgery. The FGF19 and FGF21 serum assays (pg/mL) were performed according to the manufacturer’s recommendations (BioVendor, Asheville, NC) with sample, controls, and standards assayed in duplicate. These Elisa immunoassays employed the quantitative sandwich enzyme technique which is based on polyclonal antibodies specific to the human FGF19 or FGF21. The range of detection for in serum FGF19 is 4.8–800 (pg/mL) and for FGF21 7.0–1920 (pg/mL). Samples with values outside of these ranges were omitted. The coefficient of variation (CV) values of intra-assay and inter-assay precision for FGF19 are 7% and 8.5% respectively, and for FGF21 are 3% and 9% respectively.

### Gene expression using real-time quantitative qPCR

RNA preparations from liver biospecimens taken during RYGB surgery were performed using a kit (RNeasy Lipid Tissue Mini Kit, Qiagen, Valencia, CA) and as we have previously described [10]. RNA was reverse transcribed using a high capacity cDNA reverse transcription with RNase inhibitor kit (Applied Biosystems-Life Technologies, Carlsbad, CA).

RNA quality evaluation and quantitation was performed using a Nanodrop ND-1000 spectrophotometer (Thermo Scientific, Wilmington, DE). Quantitative PCR was performed in duplicate on the ABI 7500 Fast Plate (Applied Biosystems, Life Technologies, Grand Island, NY) as we have previously described [17]. Pre-designed primers for *FGF21*, cholesterol 7 alpha-hydroxylase (*CYP7A1*), fibroblast growth factor receptor 4 (*FGF4R*), *β-Klotho*, farnesoid x receptor (*FXR*), Glycogen Synthase (*GS*), small heterodimer partner (*SHP*), hepatocyte nuclear factor 4α (*HNF4α)*, and *GAPDH* were purchased from Applied Biosystems (Hs00173927_m1, Hs00167982_m1, Hs01106908_m1, Hs00545621_m1, Hs00231968_m1, Hs00608677_m1, Hs00222677_m1, Hs00230853_m1, Hs02758991_g1, respectively). Final analysis was conducted by subtracting the Ct value of GAPDH from the Ct value of the gene of interest [40-Δ(Ct-Ct of GAPDH)] so that lower gene expression does not appear with a negative sign but rather as a shorter column in the bar graphs, relative to the control or other references as we have previously described [18].

### Statistical analyses

Means (standard deviation) and percentages were used to describe the demographics, body size, and diabetes/lipid laboratory levels of the study populations. These characteristics were compared between groups by using two-sample t-tests (continuous data) and Fisher’s exact test (categorical data). The distributions of FGF19/21 were compared between groups using nonparametric statistical tests (i.e., Wilcoxon rank sum test and Kruskal-Wallis Test) to account for the inherent heteroskedasticity of skewed distributions (as confirmed by Shapiro-Wilk test for skewness). Multiple logistic regression was used to simultaneously estimate the odds ratio for presence of diabetes for various levels of FGF19 and FGF21. To determine if there was a synergistic effect FGF19 and FGF21 on diabetes, an interaction term was added to the model.

To identify clinical factors that were associated with FGF19 and with FGF21, a PheWAS was conducted using 205 clinical variables (**Table C in S1 File**) that included 92 medication variables, 50 comorbidity variables, 45 laboratory results, 15 other various variables (e.g. demographics, anthropometrics, alcohol/smoking status) and 3 perioperative specifics. The level of association between these variables and FGF19/21 was measured using p-values resulting from Wilcoxon Rank Sum tests (for categorical clinical variables such as use of metformin) and least-squared linear regression analysis (for continuous clinical variables such as age). Bonferonni corrections were evaluated following bivariate analysis.

Pairwise comparisons against the control or between pairs of T2D groups were performed using Students T-test and Tukey’s adjustment for multiple comparisons. For patient data, analyses were repeated after subdividing the T2D group into T2D-R and T2D-NoR using one way analysis of variance (ANOVA). SAS version 9.2 was used for all statistical analysis with P-values < 0.05 considered significant.

## Results

### Comparison of FGF19 and FGF21 circulating levels between diabetic and non-diabetic patients

Subsets of No-T2D and T2D patients (Table 1) were randomly selected and further divided according to their RYGB postoperative diabetes remission status for the measurements of the FGF19/21 serum levels (**Tables A and B in S1 File**). T2D patients had significantly lower FGF19 serum levels than No-T2D patients (**Table 1**). T2D patients, on the other hand, had higher FGF21 serum levels than No-T2D patients that trended towards statistical significance (**Table 1**).

**Table 1:** Comparison of circulating levels of FGF19 and FGF21 between diabetic (T2D) and non-diabetic (No-T2D) patients.

	Diabetes Status	N	Mean	Std Dev	Median	LowerQuartile	UpperQuartile
FGF19	No-T2D	29	201.91	142.88	162.61	101.58	258.86
FGF19	T2D	46	122.92	104.13	94.04	44.73	147.04
FGF21	No-T2D	22	393.28	247.63	363.85	205.27	471.83
FGF21	T2D	46	704.51	792.91	524.44	233.31	817.93

Diabetic patients had signifciantly lower FGF19^¶^ serum levels (pg/mL), and higher (trending) FGF21^§^ serum levels (pg/mL). P-values according to the Wilcoxon Rank sum test (^¶^: P-value, 0.0036; ^§^: P-value, 0.0871).

Moreover, both the T2D-R (N = 22, mean = 119.83 pg/mL) and T2D-NoR (N = 24, mean = 125.75 pg/mL) groups of patients had signifciantly lower FGF19 serum levels than No-T2D patients (N = 29, mean = 201.90 pg/mL) (Kruskal-Wallis Chi Square Test: 8.54, P-value = 0.0139). In the case of the serum FGF21 levels, however, there were no statistically significant differences across the three groups [T2D-R: 540.55 pg/mL, T2D-NoR: 842.24 pg/mL, No-T2D: 393.28 pg/mL, (Kruskal-Wallis Chi Square Test: 4.39, P-value = 0.1111)] despite the fact that the two diabetes groups had higher FGF21 serum levels.

### Association analysis of circulating FGF19 and FGF21 with clinical variables

In order to test for the presence of an association between FGF19 and/or FGF21 serum levels with various metabolic phenotypes, we performed a PheWAS by using 205 clinical variables from EMR (**Table C in S1 File**). Results were categorized into four groups representing anthropometric, disease, diagnostic, and medication classes (**Table 2**). A total of 21 variables were signifciantly associated with either FGF19 or FGF21 but never with both. In addition, it was always higher FGF21 or lower FGF19 that were associated with some variable. Higher FGF21 levels were associated with older age higher height, and larger waist circumference (**Table 2**). The diagnosis of diabetes and liver fibrosis were associated with lower FGF19 levels, while, higher FGF21 levels were associated with hypertension. In contrast, diagnostic features of diabetes (i.e., higher glucose levels and HbA1c) were associated with higher FGF21 levels (**Table 2**). Antidiabetic medications however, with the exception of sulphonylureas, were associated with lower FGF19 levels (**Table 2**). Following Bonferroni corrections for multiple testing, only the association of high glucose with high FGF21 levels remained significant.

**Table 2:** Phenome-wide association analysis (PheWAS) of 205 clinical variables with FGF19 or FGF21 serum levels (pg/mL).

	Direction of association	FGF19(P-value)	FGF21(P-value)
Anthropometric	Older age ***x*** higher FGF21	0.11095	0.00115
	Taller ***x*** higher FGF21	0.83335	0.02166
	Larger waist ***x*** higher FGF21	0.35547	0.03535
Disease	Diabetes ***x*** lower FGF19	0.00142	0.11761
	Hypertension ***x*** higher FGF21	0.48434	0.0037
	Liver fibrosis ***x*** lower FGF19	0.00506	0.93641
Diagnostic	Higher glucose* ***x*** higher FGF21	0.15621	0.00003
	Higher HbA1c ***x*** higher FGF21	0.1995	0.00188
	Lower LDL ***x*** higher FGF21	0.46145	0.0353
	Higher creatinine ***x*** higher FGF21	0.42198	0.01965
	Lower EGFR ***x*** higher FGF21	0.84129	0.00482
Medication	Metformin ***x*** lower FGF19	0.02677	0.44619
	Sulfonylurea ***x*** lower FGF19	0.02697	0.05797
	Insulin ***x*** higher FGF21	0.87188	0.04984
	ISA ***x*** lower FGF19	0.00984	0.49681
	ACE ***x*** higher FGF21	0.66932	0.00164
	Statins ***x*** higher FGF21	0.14334	0.00295
	Multiple meds ***x*** higher FGF21	0.48933	0.00266
	CCB ***x*** higher FGF21	0.95894	0.00389
	Salicylates ***x*** higher FGF21	0.80814	0.00954
	Potassium ***x*** higher FGF21	0.7106	0.01953

P-values were according to Wilcoxon rank sum test (categorical clinical variables or least-squared linear regression analysis (for continuous clinical variables). After Bonferonni corrections for multiple testing, only the association of higher glucose* with higher FGF21 remained significant. LDL: low density lipoprotein, EGFR: estimated glomerular filtration rate, ISA: insulin sensitizing agents other than metformin, ACE: ACE inhibitors, CCB: calcium channel blockers.

### Correlation analysis between FGF19 and FGF21 circulating levels (pg/mL) for diabetic (T2D) and non-diabetic (No-T2D) patients

There was no significant correlation between FGF19 or FGF21 within No-T2D, T2D (i.e., all T2D patients in one group) groups of patients or when separating the T2D into T2D-R and T2D-NoR patients with diabetes remission after RYGB surgery.

**Fig 1:**
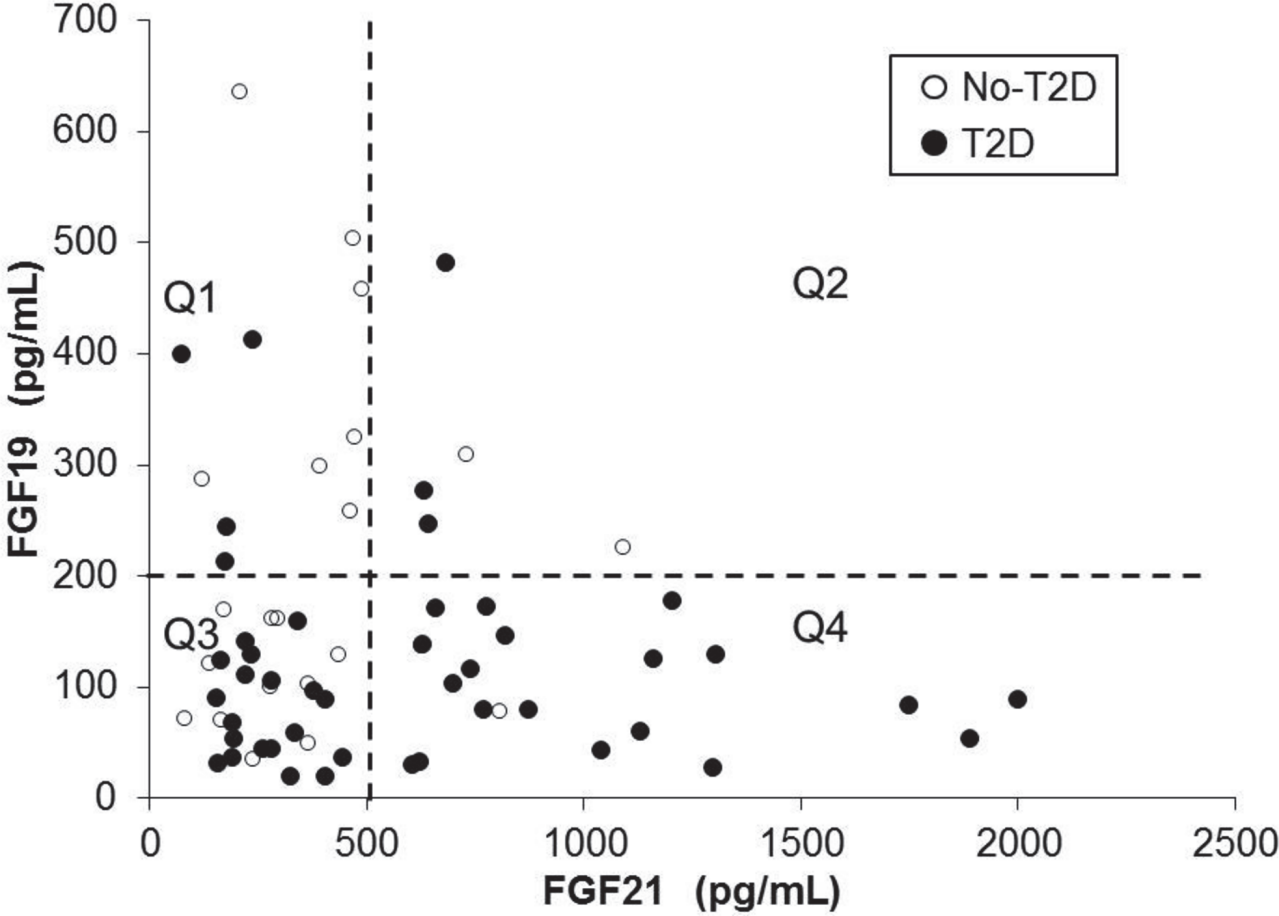
Scatter plot and correlation analysis between FGF19 and FGF21 circulating levels (pg/mL) for diabetic (T2D) and non-diabetic (No-T2D) patients. The FGF19 and FGF21 data for both T2D and No-T2D data were divided into quartiles (Q1, Q2, Q3, and Q4) by using the serum levels of 500 pg/mL for FGF21 and 200 pg/mL for FGF19 as cut offs. The following four classes of patients according to risk for having diabetes were formed: Q1, lowest risk: FGF19>200 and FGF21<500: n = 4 of 11 (36% with diabetes); Q2, low-to-moderate risk: FGF19>200 and FGF21>500: n = 3 of 5 (60% with diabetes); Q3, moderate-to-high risk: FGF19<200 and FGF21<500: n = 19 of 30 (63% with diabetes); Q4, highest risk: FGF19<200 and FGF21>500: n = 19 of 21 (91% with diabetes). The Chi-square P-value when comparing the four quartiles was significant (0.017).

Because of the high occurrence of low serum levels for FGF19 and high serum levels for FGF21 (**Fig. 1**), the FGF19 and FGF21 data were divided into quartiles (Q1, Q2, Q3, and Q4) by using the serum levels of 500 pg/mL for FGF21 and 200 pg/mL for FGF19 as cut offs (**Fig. 1**). The following four classes of patients according to risk for having diabetes were formed: Q1, lowest risk: FGF19>200 and FGF21<500: n = 4 of 11 (36% with diabetes); Q2, low-to-moderate risk: FGF19>200 and FGF21>500: n = 3 of 5 (60% with diabetes); Q3, moderate-to-high risk: FGF19<200 and FGF21<500: n = 19 of 30 (63% with diabetes); Q4, highest risk: FGF19<200 and FGF21>500: n = 19 of 21 (91% with diabetes). The Chi-square P-value when comparing the four quartiles was significant (0.017).

Logistic regression to simultaneously estimate the odds ratios of FGF19 and FGF21 for diabetes (i.e. to determine if they were acting independently and/or test for a synergistic effect), showed that both FGF19 < 200 (OR = 3.73, 95% CI = [1.08, 12.84], p = 0.037) and FGF21 > 500 (OR = 4.28, 95% CI = [1.20, 15.27], p = 0.025) were independently associated with diabetes, but the interaction (i.e. synergistic effect) was not significant (p = 0.594).

### Comparison of hepatic gene expression between diabetic (T2D) and non-diabetic (No-T2D) patients undergoing RYGB surgery

**Fig 2:**
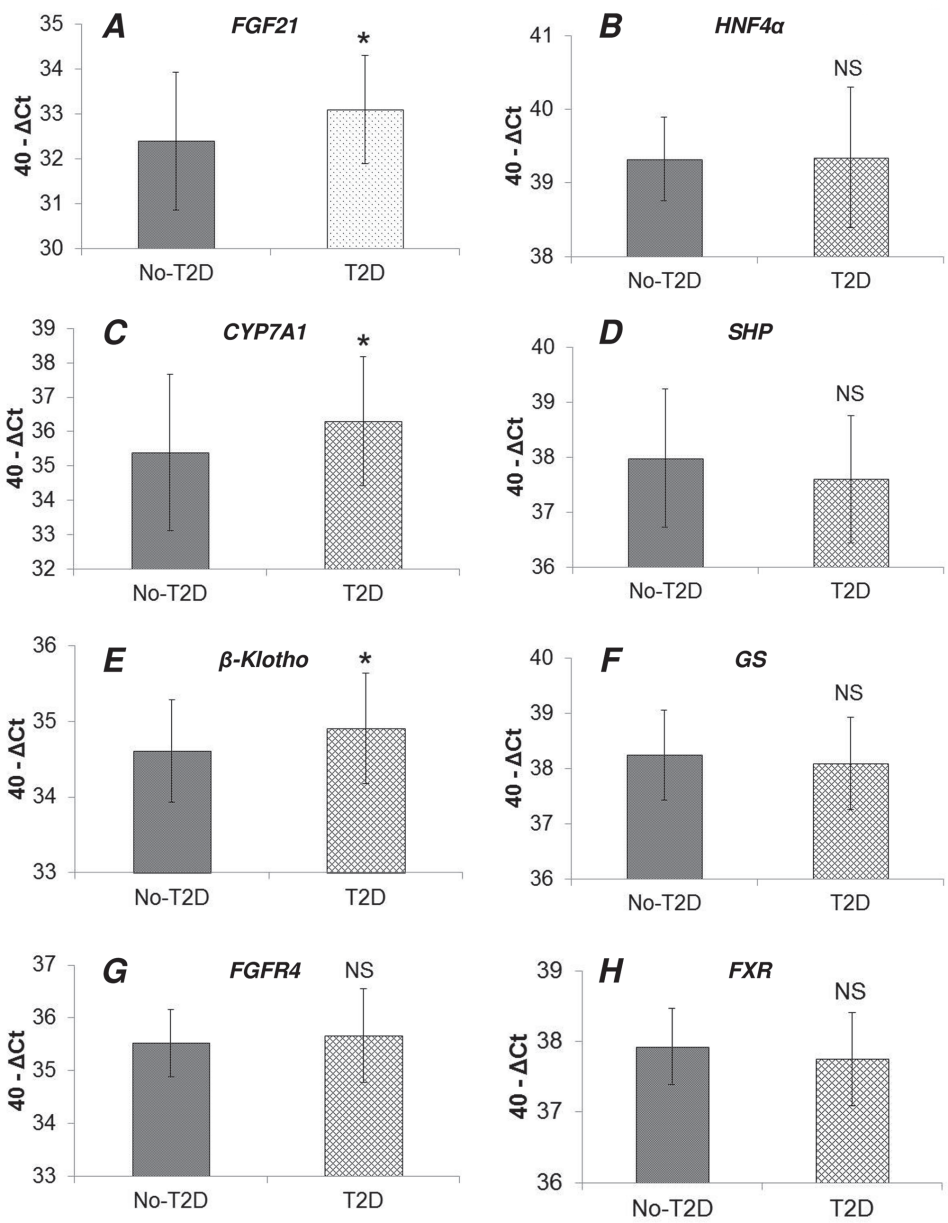
Comparison of hepatic gene expression between non-diabetic (No-T2D) and diabetic (T2D) patients. Gene expression levels (mRNA determined by real time qPCR) for genes modulating the bile acids-FGF19-FGF21 pathway in the liver were compared between non diabetic and diabetic patients. mRNA levels of *FGF21* (**A**), *CYP7A1* (**C**), and *β-Klotho* (**E**) were significantly higher in diabetic patients. There were no significant differences between diabetic and non-diabetic patients for *HNF4α* (**B**), *SHP* (**D**), *GS* (**F**), *FGFR4* (**G**), and *FXR* (**H**). Statistical analysis was performed by using the Student’s T-test (*: P-value < 0.05). NS: not statistically significant.

To test further the hypothesis that hepatic gene expression in the FGF19-FGF21 axis is dysregulated in diabetes, we compared gene expression levels (mRNA determined by real time qPCR) between T2D and No-T2D patients (**Table A in S1 File**). We found that mRNA levels of *FGF21* (**Fig. 2A**), *CYP7A1* (**Fig. 2C**), and *β-Klotho* (**Fig. 2E**) were significantly higher in diabetic patients. There were no significant differences between diabetic and non-diabetic patients for *HNF4α* (**Fig. 2B**), *SHP* (**Fig. 2D**), glycogen synthase (*GS*) (**Fig. 2F**), *FGFR4* (**Fig. 2G**), and *FXR* (**Fig. 2H**).

**Fig 3:**
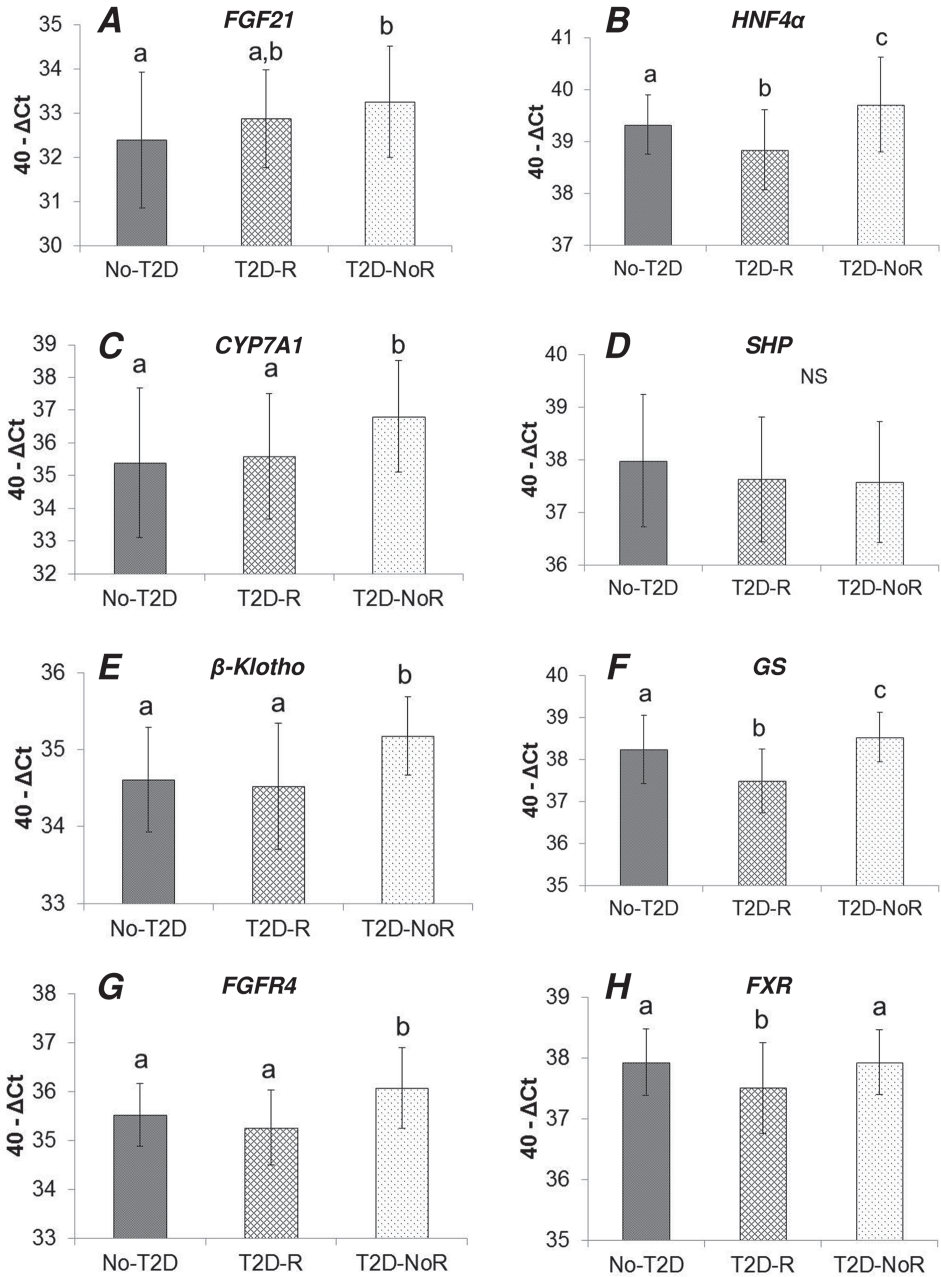
Comparison of hepatic gene expression between non-diabetic (No-T2D) and diabetic patients experiencing diabetes remission (T2D-R) or not experiencing diabetes remission (T2D-NoR) after RYGB surgery. Gene expression levels (mRNA determined by real time qPCR) for genes modulating the bile acids-FGF19-FGF21 pathway in the liver were compared between the three aforementioned groups. In the cases of *FGF21* (**A**), *HNF4α* (**B**), *CYP7A1* (**C**), *β-Klotho* (**E**), *GS* (**F**), and *FGFR4* (**G**), the T2D-NoR group of patients displayed signifciantly higher expression levels compared to control (i.e., No-T2D) and/or the T2D-R groups. There were no significant differences between the three groups for *SHP* (**D**), while, the T2D-R group had signifciantly lower expression levels for *HNF4α* (**B**), *GS* (**F**), and *FXR* (**H**), compared to No-T2D and T2D-NoR. Statistical analysis was performed by using ANOVA followed by Tukey’s test. The different letters (a, b, c) above the bars indicate statistically different groups (significance level at P-value < 0.05). Share of the same letter between groups (bars) indicates lack of statistical significance. NS: not statistically significant.

T2D patients were further stratified according to their remission status after RYGB surgery. Gene expression was thus compared between three groups: No-T2D, T2D-R, and T2D-NoR (**Table B in S1 File**). In the cases of *FGF21* (**Fig. 3A**), *HNF4α* (**Fig. 3B**), *CYP7A1* (**Fig. 3C**), *β-Klotho* (**Fig. 3E**), *GS* (**Fig. 3F**), and *FGFR4* (**Fig. 3G**), the T2D-NoR group of patients displayed significantly higher expression levels compared to No-T2D and/or the T2D-R groups. There were no significant differences between the three groups for *SHP* (**Fig. 3D**), while, the T2D-R group had signifciantly lower expression levels for *HNF4α* (**Fig. 3B**), *GS* (**Fig. 3F**), and *FXR* (**Fig. 3H**), compared to No-T2D and T2D-NoR.

## Discussion

FGF19 and FGF21 are enterohepatic hormones that play important roles in the pathophysiology of diabetes [19]. FGF19 serum levels are significantly lower in Class III obese patients with diabetes as well as in typical diabetic patients with mean BMI of 30 kg/m^2^ [10]. Moreover, FGF19 serum levels increase following RYGB surgery and particularly more so in patients that experience remission of diabetes [10]. FGF21 serum levels, on the other hand, are already higher in diabetic patients and increase further after RYGB surgery [20].

Here, we confirmed that FGF19 serum levels were lower and FGF21 higher in T2D patients compared to No-T2D patients. In line with the higher FGF21 serum levels, FGF21 mRNA levels were also higher in the livers of T2D patients. In addition, *CYP7A1* and *β-Klotho* mRNA levels were higher in T2D patients, confirming past data [10], and further underscoring the presence of dysregulation in enterohepatic signaling involving the FGF19-CYP7A1-BA pathway and FGF21 production in diabetic patients.

In order to delineate the type of diabetic patient that presents the most significant hepatic dysregulation, T2D patients were stratified according to their remission status after RYGB surgery. A common pattern emerged whereby patients that do not remit diabetes (T2D-NoR) displayed signifciantly higher expression levels for *FGF21*, *HNF4α*, *CYP7A1*, *β-Klotho*, *GS*, and *FGFR4*, compared to the No-T2D and T2D-R groups. This additional analysis provided further confirmation that the FGF19-CYP7A1-BA pathway and FGF21 hepatic expression as well as its circulating levels are indeed dysregulated in diabetes but mostly in patients with severe diabetes. We have reported that such patients manage their diabetes by using insulin-sensitizing agents other than metformin and are also commonly prescribed insulin [21].

In an effort to identify other conditions that may be associated with low FGF19 and high FGF21 serum levels, we performed a PheWAS using 205 clinical variables. It is of particular interest that it was either lower FGF19 or higher FGF21 serum levels that were signifciantly associated with diabetes or cardiometabolic phenotypes but never the two together. Although this outcome could be coincidental it may also suggest the presence of a level of complementarity in the functional roles of FGF19 and FGF21, as previously suggested [12]. It should be pointed out that after applying Bonferroni corrections for multiple testing only the association between higher glucose levels and higher FGF21 levels remained statistically significant. The unadjusted PheWAS analysis, however, revealed that higher FGF21 levels were also associated with increased height, waist circumference, hypertension, high creatinine levels, the use of statins, and the use of ace inhibitors among other clinical variables. These data suggest that high preoperative FGF21 serum levels are associated with multiple components of the metabolic syndrome. This possibility is supported by a recent study whereby exogenous FGF21 had marginal effects on glucose lowering but improved HDL levels and lowered atherogenic apolipoprotein concentration [22]. We did not have available, paired, postoperative serum samples to determine whether the reported increase of FGF21 after RYGB surgery is specific to patients remitting or not remitting diabetes.

There was no significant correlation between FGF19 and FGF21 serum levels within any groups and neither was there a significant synergistic effect. There was, however, a high frequency of patients with low FGF19 and high FGF21serum levels. Similar alterations in FGF19 and FGF21 have been reported in obese patients compared to leaner controls [23]. The FGF19 and FGF21 data were thus divided into quartiles (Q1, Q2, Q3, and Q4). The high risk quartile, Q4, (i.e., FGF19 < 200 and FGF21 > 500) determined that 91% of patients had diabetes. This outcome could be used for identifying patients with high probability for having diabetes.

In conclusion, higher levels of circulating FGF21 were associated with diabetes and various cardiometabolic disease phenotypes. When combining high FGF21 with low FGF19 levels, 91% of Class III obese patients had diabetes. Moreover, hepatic expression levels of *FGF21*, *CYP7A1*, *HNF4α*, *β-Klotho*, *FGFR4*, *and GS* were higher in T2D patients that do not remit diabetes after RYGB surgery. Taken together, these data provide evidence that FGF21 and the FGF19-BA pathway are significantly perturbed in severe diabetes and could represent suitable targets for the development of a new class of treatments for diabetes provided that the pro-oncogenic effects of FGF19 could be curtailed [24,25]. Prospective clinical trials using pharmacologic and/or nutritional interventions that can modulate circulating FGF19 and FGF21 levels could help elucidate further the exact roles of these two hormones in the pathophysiology of diabetes.

## Supporting Information

S1 File With data on the basic characteristics of the study cohorts (Tables A and B), and the 205 clinical variables that were used for the PheWAS (Table C), are provided. (DOCX)
