# Supplementary material for: Perturbations of Fibroblast Growth Factors 19 and 21 in Type 2 Diabetes
Source: PLoS One. 2015 Feb 9;10(2):e0116928. doi: 10.1371/journal.pone.0116928 (PMC4321834; doi:10.1371/journal.pone.0116928)
Supplement: S1 File — (DOCX) [file pone.0116928.s001.docx]

**SUPPORTING INFORMATION (SI) FILE**

**Title**: Perturbations of fibroblast growth factors 19 and 21 in type 2 diabetes

**Authors**: Stephen L. Roesch^1^, Amanda M. Styer^1^, G. Craig Wood^1^, Zachary Kosak^1^, Jamie Seiler^1^, Peter Benotti^1^, Anthony T. Petrick^2^, Jon Gabrielsen^2^, William E. Strodel^2^, Glenn S. Gerhard^1,3^, Christopher D. Still^1^, and George Argyropoulos^1^,*.

**Affiliation**: ^1^Institute of Obesity, Geisinger Health System, Danville, PA, ^2^Department of Surgery, Geisinger Health System, Danville, PA, ^3^Department of Biochemistry and Molecular Biology and Department of Pathology and Laboratory Medicine, Pennsylvania State University, Hershey, PA.

*: Corresponding author: gargyropoulos1@geisinger.edu

***Table A***. **Descriptive characteristics of study cohorts divided according to their preoperative status of diabetes (SD: Standard deviation)**.

|  | **No-Diabetes**  **(No-T2D)**  **N=66** | **Diabetes**  **(T2D)**  **N=62** | **P-value** |
| --- | --- | --- | --- |
| Age (years),  mean (SD) | 40.1 (8.6) | 50.6 (10.6) | **<0.0001^1^** |
| Female  (%) | 89% | 79% | 0.107^2^ |
| White race  (%) | 95% | 100% | 0.245^3^ |
| Height (cm),  mean (SD) | 165.0 (8.2) | 164.9 (8.9) | 0.973^1^ |
| Weight (kg),  mean (SD) | 136.8 (31.6) | 134.3 (25.0) | 0.620^1^ |
| BMI (kg/m^2^),  mean (SD) | 50.0 (9.4) | 49.3 (8.0) | 0.653^1^ |
| Glucose (mg/dL),  mean (SD) | 85.7 (7.6) | 130.4 (68.0) | **<0.0001^1^** |
| Insulin (µU/mL),  mean (SD) | 18.4 (13.3) | 22.2 (16.9) | 0.163^1^ |
| HbA1c (%),  mean (SD) | 5.4 (0.2) | 7.3 (1.3) | **<0.0001^1^** |
| LDL (mg/dL),  mean (SD) | 112.5 (30.3) | 90.5 (34.9) | **0.0003^1^** |
| HDL (mg/dL),  mean (SD) | 48.3 (12.6) | 43.7 (8.6) | **0.016^1^** |
| Cholesterol (mg/dL),  mean (SD) | 188.7 (37.5) | 169.3 (41.3) | **0.0062^1^** |
| Triglycerides (mg/dL),  mean (SD) | 139.2 (77.3) | 181.4 (96.4) | **0.0070^1^** |
| ^1^T-test, ^2^Chi-square test, ^3^Fisher’s exact test | | | |

***Table B*.** **Descriptive characteristics of study cohorts divided according to their diabetes remission status after Roux-en-Y gastric bypass (RYGB) surgery (SD: standard deviation)**.

|  | **No-Diabetes**  **(No-T2D)**  **(N=66)** | **Diabetes with remission**  **(T2D-R)**  **(N=26)** | **Diabetes without remission**  **(T2D-NoR)**  **(N=36)** | **P-value** |
| --- | --- | --- | --- | --- |
| Age (years),  mean (SD) | 40.1 (8.6) | 45.8 (11.4) | 54.1 (8.6) | **<0.0001^1^** |
| Female  (%) | 89% | 81% | 78% | 0.259^2^ |
| White race  (%) | 95% | 100% | 100% | 0.430^3^ |
| Height (cm),  mean (SD) | 165.0 (8.2) | 165.3 (9.2) | 164.6 (8.8) | 0.952^1^ |
| Weight (kg),  mean (SD) | 136.8 (31.6) | 138.1 (29.1) | 131.5 (21.7) | 0.593^1^ |
| BMI (kg/m^2^),  mean (SD) | 50.0 (9.4) | 50.5 (9.4) | 48.5 (6.8) | 0.595^1^ |
| Glucose (mg/dL),  mean (SD) | 85.7 (7.6) | 116.2 (43.0) | 140.7 (80.5) | **<0.0001^1^** |
| Insulin (µU/mL),  mean (SD) | 18.4 (13.3) | 24.5 (15.7) | 20.6 (17.8) | 0.235^1^ |
| HbA1c (%),  mean (SD) | 5.4 (0.2) | 6.8 (1.1) | 7.7 (1.4) | **<0.0001^1^** |
| LDL (mg/dL),  mean (SD) | 112.5 (30.3) | 103.3 (34.1) | 81.1 (32.9) | **<0.0001^1^** |
| HDL (mg/dL),  mean (SD) | 48.3 (12.6) | 43.5 (10.3) | 43.8 (7.4) | 0.059^1^ |
| Cholesterol (mg/dL),  mean (SD) | 188.7 (37.5) | 176.2 (42.1) | 164.3 (40.5) | **0.012^1^** |
| Triglycerides (mg/dL),  mean (SD) | 139.2 (77.3) | 154.5 (83.0) | 200.8 (101.6) | **0.0031^1^** |
| ^1^ANOVA, ^2^Chi-square test, ^3^Fisher’s exact test | | | | |

***Table C*. List of the 205 clinical variables that were used in the phenome-wide association analysis (PheWAS).**

***Baseline information and demographics (n=15)***

1. Age
2. Alcohol use
3. BMI at baseline
4. Days in pre-operative program
5. Ethnic group
6. Gender
7. Height
8. Number of co-morbidities (from list below)
9. Number of medications (from list below)
10. Preoperative weight loss
11. Rating from initial dietician evaluation (red, yellow, green)
12. Rating from initial psychological evaluation (red, yellow, green)
13. REE
14. Smoker
15. Waist circumference

***Comorbidities on problem list from baseline to surgery using 3-digit ICD, codes (n=50)***

- 1. ICD_244
  2. ICD_250
  3. ICD_251
  4. ICD_256
  5. ICD_268
  6. ICD_269
  7. ICD_272
  8. ICD_274
  9. ICD_276
  10. ICD_296
  11. ICD_300
  12. ICD_305
  13. ICD_307
  14. ICD_309
  15. ICD_311
  16. ICD_327
  17. ICD_346
  18. ICD_354
  19. ICD_357
  20. ICD_401
  21. ICD_414
  22. ICD_427
  23. ICD_428
  24. ICD_429
  25. ICD_477
  26. ICD_493
  27. ICD_530
  28. ICD_553
  29. ICD_564
  30. ICD_592
  31. ICD_625
  32. ICD_626
  33. ICD_627
  34. ICD_715
  35. ICD_719
  36. ICD_722
  37. ICD_724
  38. ICD_726
  39. ICD_728
  40. ICD_729
  41. ICD_780
  42. ICD_782
  43. ICD_784
  44. ICD_785
  45. ICD_786
  46. ICD_787
  47. ICD_789
  48. ICD_790
  49. ICD_794
  50. ICD_796

***Medications on reconciliation list from baseline to 1 month prior to surgery using subclass (n=92)***

- 1. ACE
  2. Aminopenicillins
  3. AnalgesicCombo
  4. AnalgOther
  5. AnorexNonAmph
  6. AntiadrenergicAntiHTN
  7. AntianxietyAgentsMisc
  8. AnticonvMisc
  9. AnticonvulsantsBenzod
  10. AntidepressMisc
  11. AntiemetAnticholinergic
  12. AntifungalsTopical
  13. AntihistNonsed
  14. AntihistPhenothiazines
  15. AntiHTNcomb
  16. AntihyperlipidCombo
  17. AntiInfectiveAgentsMisc
  18. AntiInfectiveMiscCombo
  19. AntiObesityAgents
  20. AntiparkinDopaminergic
  21. Antitussives
  22. ARB
  23. Azithromycin
  24. BBCS
  25. BBNS
  26. Benzod
  27. Biguanides
  28. BronchoAnticholinergics
  29. BulkLaxatives
  30. Calcium
  31. CCB
  32. Cephalosporins1stGen
  33. Clarithromycin
  34. Cobalamins
  35. ComboContraceptOral
  36. CorticTopical
  37. Cough_Cold_AllComb
  38. Coumarin
  39. Dibenzapines
  40. DiureticCombo
  41. Estrogens
  42. FibricAcid
  43. Fluoroquinolones
  44. FolicAcidFolates
  45. GastrointestlStimulants
  46. Glucocort
  47. GoutAgents
  48. H2
  49. Heparin
  50. ImidazoleRelatedAntifungal
  51. IncretinMimetAgGLP1
  52. InsSensAgents
  53. Insulin
  54. IntestCholAbsorptionInhib
  55. Iron
  56. LeukotrieneModulators
  57. LoopDiur
  58. ModifiedCyclics
  59. MultiVit
  60. MultiVitMinerals
  61. MuscleRelax
  62. NasalSteroids
  63. Nitrates
  64. NonBarbitHypnotics
  65. NSAID
  66. OilSolVit
  67. OpioidAgonist
  68. OpioidCombo
  69. PenicillinCombo
  70. PlateAggrInhibitors
  71. Potassium
  72. PotassiumSparingDiuretics
  73. PPI
  74. ProgestContratInjectable
  75. Progestins
  76. Salicylates
  77. SerotoninAgonists
  78. SmokingDeterrents
  79. SNRI
  80. SSRI
  81. Statins
  82. SteroidInhalants
  83. Sulfonylureas
  84. SurfactantLaxatives
  85. Sympath
  86. Tetracyclines
  87. Thiazides
  88. ThyroidHorm
  89. Tricyclic
  90. UlcerTherapyCombo
  91. UrinaryAntispasmodics
  92. WaterSolubleVitamins

***Labs prior to surgery found in >50% of population (n=45)***

- 1. AlkPhos
  2. ALT
  3. AnionGap
  4. AST
  5. bun
  6. calcium
  7. CHLORIDE
  8. CHOL
  9. CholHDLRat
  10. CO2
  11. creat
  12. Creatinine, RD Urine
  13. Ferritin
  14. FolicAcid
  15. GFR
  16. glucose
  17. hba1c
  18. HCT
  19. HDL
  20. HEMOGLOBIN
  21. IBC
  22. insulin
  23. Iron
  24. LDL
  25. MCH
  26. MCHC
  27. MCV
  28. MPV
  29. PLATELET
  30. potassium
  31. Prot/Creat Ratio
  32. Protein, RD Urine
  33. PTH
  34. RBC
  35. RDW
  36. sodium
  37. TotBili
  38. TransSat
  39. TRIG
  40. TSH
  41. Vitamin D 25 OH D2
  42. Vitamin D 25 OH D3
  43. Vitamin D 25 TOTAL
  44. WBC
  45. Zinc levels

***Perioperative information and liver pathology (n=3)***

- 1. Access (Laparoscopic versus Open)
  2. Steatosis
  3. Any fibrosis
